# The association of HIV and easy access to narcotics in Pakistan; calling drug policy makers

**DOI:** 10.1186/s40545-019-0199-5

**Published:** 2019-12-18

**Authors:** Tauqeer Hussain Mallhi, Yusra Habib Khan, Amjad Khan, Nasser Hadal Alotaibi, Abdulaziz Ibrahim Alzarea, Furqan Khurshid Hashmi

**Affiliations:** 1 Department of Clinical Pharmacy, College of Pharmacy, Jouf University, Sakaka, Al-Jouf Kingdom of Saudi Arabia; 2 Department of Pharmacy, Quaid-i-Azam University, Islamabad, 45320 Pakistan; 3 University College of Pharmacy, University of the Punjab, Allama Iqbal Campus, Lahore, 54000 Pakistan

**Keywords:** HIV, Schedule G, Drug act, Drug policy, Narcotics abuse, PWID, Pakistan, Drug act

## Abstract

HIV in Pakistan is concentrated to people who inject drugs (PWID) and easy accessibility of narcotics to this population cannot be disregarded as a risk factor of growing encumbrance of AIDS in the country. All the narcotics and other medications having high potential of abuse are stratified into Schedule G of Punjab Drug Rules 2017. According to these rules, drugs in Schedule G shall be sold in pharmacy under the direct supervision of qualified pharmacist. However, Schedule G is not implemented in Punjab due to continuous resistance from pharmaceutical stakeholders including medical store owners (who are barred to sell drugs from schedule G). Since 1.6 million PWID reported misuse of prescription opioids for non-medical use, delayed implementation of schedule G is attributing to the unabated sale of narcotics without prescription and for non-medical purposes, which is further contributing to the staggering number of PWID in the country. Implementing schedule G will not only curb the existing situation of HIV but will also mitigate the contribution of PWID towards the future events.

**Dear Editor,**

In 2019, an unprecedented number of HIV positive cases have been recorded in Pakistan (Fig. 1). Several health experts across the country reported the outbreak and underscored various contributing factors of this menace [1–4]. A recent rural epidemic of HIV in Sindh province of Pakistan is reported to be caused by poor disease awareness and literacy in rural community, use of contaminated syringe by a quack impersonating a doctor and limited coverage of national AIDS control program (NACP) [2]. Similar outbreak has been experienced in Punjab province, where prevalence of HIV hiked from 1.29 to 13.38% in Kot Imrana (a small village in Punjab) during a brief period of 6 months. Most important drivers of this outbreak were the unsafe use of syringes through quackery followed by poor coordination among healthcare departments [1]. Other factors linked to the current HIV situation in Pakistan are unsafe blood transfusion, reuse of needles, male circumcision with unhygienic blades, and ear and nose piercing with unsafe needle. The contribution of these factors to the HIV epidemics is alarming and we urge NACP, World Health Organization (WHO) and UNAIDS to address these momentous issues.

**Fig. 1:**
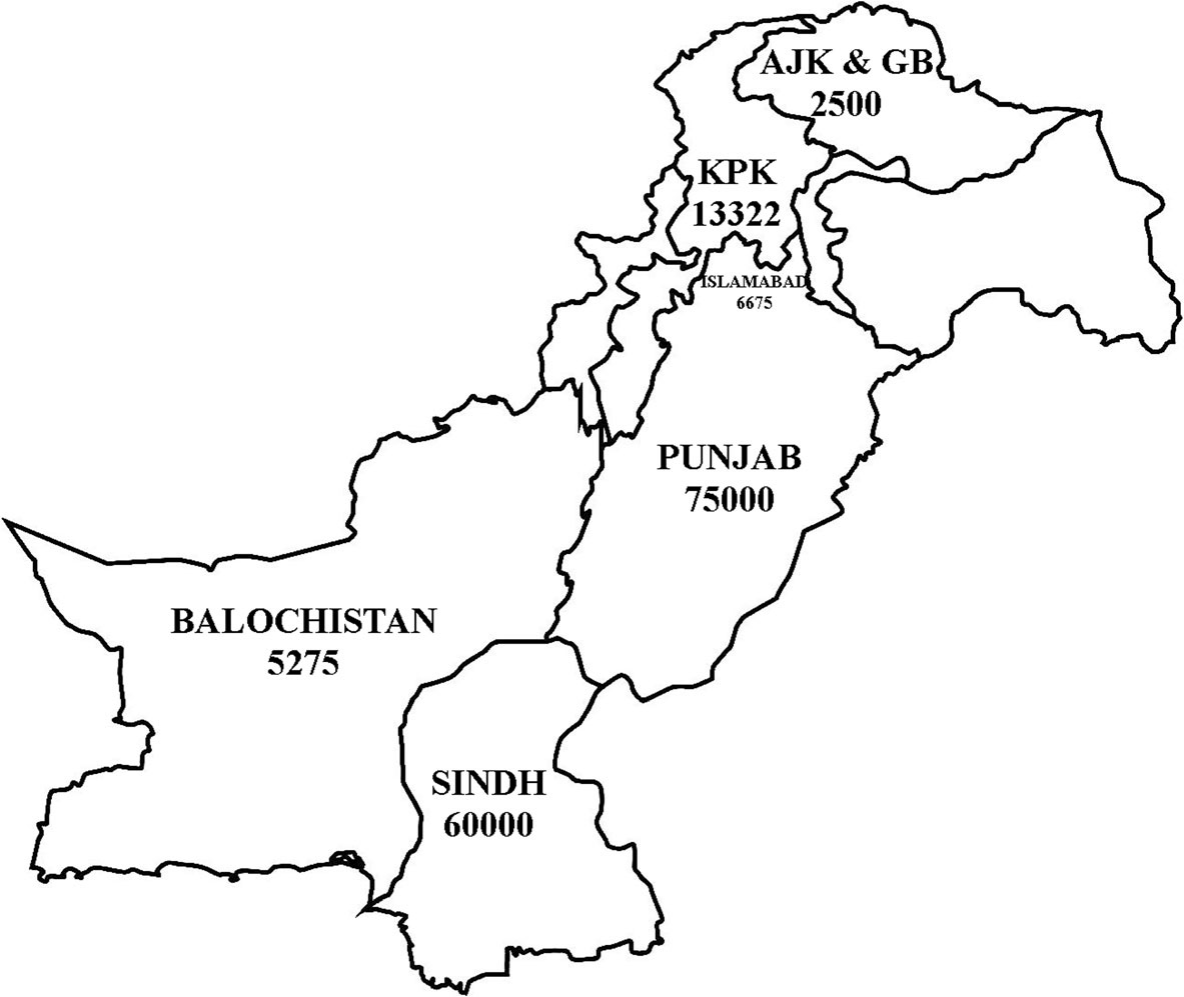
Confirmed Cases of HIV in Pakistan (AJK & GB: Azad Jammu and Kashmir & Gilgit-Baltistan, KPK: Khyber Pakhtunkhwa), Source: Reference [16]

It is pertinent to mention that HIV epidemics in Pakistan are primarily concentrated to the people who inject drugs (PWID), accounting approximately 38% of currently registered patients [1, 5]. Since PWID pose themselves to the imminent risks of contracting HIV^6^, we felt inclined to share that accessibility of injectables (i.e. narcotics) to this population should also be considered among causes of HIV and must be addressed by the Government of Pakistan in haste to quell the further disease spillover. In all previous reports, this factor is neglected by health professionals; thereby we took an opportunity to demonstrate the relationship of uncontrolled sale of narcotics/opioids and recent HIV outbreak in Pakistan.

PWID are driving HIV epidemic in Pakistan since last decade and count substantial proportion of HIV patients currently registered and receiving antiretroviral therapy (ART) [1, 5–7]. Due to its large share of the population; Punjab has the highest number of drug users with approximately 260,000 PWID. HIV prevalence among PWID has steadily increased from 10.8% in 2005 to 27.2% in 2011. Alarmingly, several cities in Pakistan reported < 40% prevalence of HIV among PWID, including Faisalabad (52.5%), D.G. Khan (49.6%), Gujrat (46.2%), Karachi (42.2%) and Sargodha (40.6%). The latest round of Integrated Biologic and Behavioral Surveillance Survey (IBBS) indicated a weighted prevalence of PWID as 36.8% among ten cities of Punjab [8]. Based on a model by Reza et al., the total number of PWID infected with HIV may reach 68,000 by 2020 [9]. A report of UN office of Drugs and Crimes (UNODC) concludes that about 6.7 million drug users have easy access to opiates in Pakistan [10–12]. Of these, nearly 1.6 million people reported misuse of prescription opioids for non-medical use [11]. A recent estimate of NACP concluded that preferred drugs of choice for PWID in Pakistan are AVIL® (injection containing antihistamine pheneramine maleate) and heroin. This report evaluated the pattern of drug use among PWID from 14 cities of Pakistan and found the use of several pharmaceutical products including Valium® (Diazepam), Phenergan® (Promethazine) and Restoril® (Temazepam) [8]. These findings underscore that substantial proportion of PWID has access to the prescription narcotics in the country. Despite these upsetting figures, delayed implementation of schedule G is further facilitating the access to these dangerous drugs, contributing to the staggering number of PWID in the country.

There are two types of drug sale outlets in Pakistan; Pharmacy (Category A: operated under the direct supervision of qualified pharmacist using license Form-9) and Medical Store (Category B: operated by non-pharmacist individuals under license Form-10). According to the Punjab Drug Rules 2007 under section 44 of Drugs Act 1976, around 145 lifesaving and vital medications are stratified into Schedule G. All narcotics and other medications having high potential of drug abuse are included in Schedule G (Additional file 1). These rules barred licensees of medical store (category B) from selling medications from schedule G [13]. However, schedule G was not implemented in the province and allowed grace period of 10 years following the severe resistance and series of protests by licensees or owners of medical stores. Recently in 2017, Government of Punjab amended the rules by implementing the schedule G and restricted the sale of narcotics by pharmacies under the supervision of qualified pharmacists. But the conditions did not differ much from the last incident and these amendments caused worst shutter down strike from chemists, medical store retailers and manufacturers across the province. Subsequently, these amendments have been withdrawn and further grace period of six years (till 2023) was agreed for the implementation of schedule G [14]. Since majority of the salespersons working on medical stores did not even attend college and lack formal education on drug use, continuous relaxation of schedule G is attributing to the uncontrolled sale of opiates/narcotics without any prescription and for non-medical purposes.

On the other hand, most of the pharmacies in Pakistan are running unauthorized on rented drug sale license in the absence of qualified pharmacists. These pharmacies are managed by a diverse group of dispensers having no authority to sell medications. Though Pharmacy Council and Court of Law has directed that renting pharmacy registration is a crime but practice is rampant in connivance with officials of health departments [15]. This malpractice is further promoting the unabated sale, availability and accessibility of opiates/narcotics and other controlled substances for non-medical purposes.

We believe that growing encumbrance of AIDS in the country is also associated with uncontrolled sale and access of injections to PWID. These factors tend to surge the proportion of PWID not only in Punjab but also in other provinces of Pakistan, thereby facilitating the risks of HIV in the country. Since considerable contribution of PWID in the existing situation of HIV and poor coverage of AIDS control program to this population cannot be disregarded, there is dire need of legislative and control strategies to prevent the unabated sale of prescription drugs to PWID. Implementing schedule G, ensuring physical appearance of pharmacists during drug sale process, fostering the prescription sale of narcotics and crackdown against illicit sale of drugs to PWID will not only assist NACP to curb the existing situation but will also mitigate the contribution of PWID towards the future events. It is important to mention that schedule G will only preclude the irrational sale of drugs to PWID while accessibility of street drugs from illicit markets will remain unaffected. Ministry of Health can play a crucial role by constructing a national body consisting of representatives from NACP and Drug Regulatory Authority of Pakistan (DRAP) to mitigate the hazards of PWID involvements in HIV epidemics. We strongly believe that collaborative and coordinated maneuvers between DRAP, NACP and provincial primary/secondary healthcare departments (P&SHC) would have synergetic impact and could go a long way in combating the AIDS in Pakistan. Moreover, it is of utmost importance to explore the sources of drug supply to PWID in Pakistan through well-structured studies.

## Supplementary information

**Additional file 1.** Schedule G under Punjab Drug Rules 200.

## Data Availability

Not Applicable.
