# Supplementary material for: The association of HIV and easy access to narcotics in Pakistan; calling drug policy makers
Source: J Pharm Policy Pract. 2019 Dec 18;12:37. doi: 10.1186/s40545-019-0199-5 (PMC6918682; doi:10.1186/s40545-019-0199-5)
Supplement: Supplementary file 1 — Additional file 1. Schedule G under Punjab Drug Rules 200. [file 40545_2019_199_MOESM1_ESM.pdf]

All records and register shall be maintained in accordance with the laws in force

Any container taken from the poison cup board shall be replaced therein immediately after use and the cupboard locked. The keys of the poison cupboard shall be kept in the personal custody of the responsible person.

Drugs when supplied shall have labels conforming to the provisions of laws in force.

Note; The above requirements are subject to modification or the directions of the Licensing Authority, if the Authority is of the opinion that having regards to the nature of drugs dispensed, compounded or prepared by the licensee it is necessary to relax the above requirements in the circumstances of a particular case.

================================

Schedule G

[See rule 20(1)(e)]

DRUGS NOT TO BE SOLD/STORED BY LICENCEE IN FORM NO.10

1. **Antileprosy**

i	Rifampicin Injection	iv	Ethionamide
ii	Dapsone	v	Prothionemide
iii	Clofamazine		

2. **immunological products, Vaccines, Sera / Anti Sera**

i	Anthrax Vaccine	ix	Rubella Vaccine
ii	BCG Vaccine	x	Pneumococcal vaccine
iii	Botulisms Antitoxin	xi	Poliomyelitis Vaccine
iv	Cholera Vaccine	xii	Smallpox Vaccine
v	Diphtheria Vaccine	xiii	Typhoid Vaccine
vi	Influenza Vaccine	xiv	Immunoglobulins
vii	Measles Vaccine	xv	Rabies Vaccine
viii	MMR Vaccine	xvi	Homophiles Influenza-Type B Vaccine

3. **Products Related with Malignant Diseases and Immunosupression**

i	Folinic Acid	xiii	Mitozantrone
ii	Doxorubicin HCl	xiv	Methotrexate
iii	Mercaptopurine	xv	Vinblastine
iv	Thioguanine	xv	Carboplatin
v	Vincristine	xvii	Bleoimycin
vi	Cisplastin	xviii	Dactinomycin
vii	Busulphan	xix	Chlorambucil
viii	Carmustine	xx	Dacarbazine
ix	Lomustine	xxi	Amasascrine
x	Cyclophosphamide	xxii	Azathioprine
xi	Melphalan	xxiii	Cyclosporin etc
xii	Fluorouracil		

4. **Drugs of Anesthesia and Inhalation Anesthetics**

i	Propofol	viii	Mitazolam
ii	Enfluran	ix	Naloxone Hcl
iii	Isofluran	xv	Vancuronium
iv	Halothane	xi	Pancuronium
v	Bupivacain	xii	Tubocuraine
vi	Thiopentone	xiii	Suxamethonium
vii	Benzodiazepine	xiv	Neostigmine

5. Antibiotics

i	Spectinomycin	ii	Vancomycin
iii	Teicoplanon	iv	Colistin
v	Sodium Fusidate	vi	Imipenem

6**. Inotropics**

i	Primacor	ii	Milrinone
iii	Enoximone		

7. Injection Prostaglandins

i	Dinoprostone	ii	Carboprost
iii	Gemeprost		

8. Alpha Blocker

i	Prazosin HCl	ii	Indoramine
iii	Daxazosing	iv	Alfuzosin

9. **Biotechnological Products**

i	Interferon	ii	Erythropoetin

10. **Narcotics, Psychotropic / Tri Cyclic Anti Depressant**

i	Morphine	xviii	Chlorpromazine
ii	Buprenorphine	xix	Meprobamate
iii	Nalbuphine	xx	Chlordiazepoxide
iv	Fantanil	xxi	Alprozolam
v	Pethidine	xxii	Clonazepam
vi	Lorazepam	xxiii	Flurazepam
vii	Temazepam	xxiv	Loprazolam
viii	Oxazepam	xxv	Dothiepin
ix	Amoxapine	xxvi	Doxepin
x	Iprine Dole Codine	xxvii	Nortriptyline
xi	Pentazocine	xxviii	Trimipramine
xii	Phenelzine	xxix	Tranycypromine
xiii	Lithium	xxx	Flupenthixol
xiv	Dextropropoxyphene	xxxi	Tryptophan
xv	Clomipramine	xxxii	Imipramine
xvi	Mianserin	xxxiii	Amipriptyline etc
xvii	Maprotiline		

11**. Antiviral**

i	Acyclovir	Vii	Idoxuridine
ii	Amantadine HCl	viii	Ribavirin
iii	Famciclovir	ix	Vidarabin
iv	Inosine Pranolsex	x	Trifluridine
v	Zidovudine	xi	Methisozone etc
vi	Ganciclovir		

12. **Thrombolytic Enzymes**

i	Alteplase	ii	Anislreplase
iii	Streptokinase	iv	Urokinase

13. **Product Used in Dialysis**

i	Peritoneal Dialysis & Haemodialysis	ii	Lysine Solution (Irrigation Solution)
iii	Hyper tonic Solution	iv	Isotonic Solution

14. **Creams and aerosols Steroidal Preparations**

i	Prednisolone	ii	Methylprednislone
iii	Tramcionolone	iv	Dexamethasone
v	Beclomethasone	vi	Hydrocortisone

15. **Hormones**

i	Vasopressin	vi	Finasteride

ii	Desmopressin	vii	Finasteriode
iii	Stanozolol	viii	Somatropin
iv	Nandrolone	ix	Testosterone
v	Mesterolone	x	Progestrogens
